# School performance is impaired in children with both simple and complex congenital heart disease

**DOI:** 10.3389/fped.2023.1073046

**Published:** 2023-02-23

**Authors:** Camilla Omann, Rasmus Kristensen, Ann Tabor, J. William Gaynor, Vibeke E. Hjortdal, Camilla Nyboe

**Affiliations:** ^1^ Department of Cardiothoracic & Vascular Surgery, Aarhus University Hospital, Aarhus, Denmark; ^2^ Department of Clinical Medicine, Aarhus University, Aarhus, Denmark; ^3^ Department of Cardiothoracic Surgery, Copenhagen University Hospital–Rigshospitalet, Copenhagen, Denmark; ^4^ Department of Clinical Medicine, Copenhagen University Hospital, Copenhagen, Denmark; ^5^ Center of Fetal Medicine, Department of Obstetrics, Copenhagen University Hospital–Rigshospitalet, Copenhagen, Denmark; ^6^ Division of Cardiothoracic Surgery, Children’s Hospital of Philadelphia, Philadelphia, PA, United States

**Keywords:** congenital heart disease, school performance, neurodevelopment, special education, preeclampsia, maternal smoking

## Abstract

**Background:**

We do not know if children born with a simple or uncorrected congenital heart disease (CHD) have school performance issues and an increased need for special education compared to healthy peers. With this study we examine the school performance and the need for special education in children with both simple and complex CHD. Further, we evaluate if exposure to preeclampsia or smoking affects the need for special education.

**Methods:**

In this nation-wide population based registry study, we included all Danish children with CHD born 1994–2012. In addition ten age and gender matched control per CHD child were included. Non-singletons and children born with a syndrome were excluded. Exposure was defined as having a CHD and the outcome was defined as needing special education service in the Danish primary and lower secondary school.

**Results:**

The population consisted of 7,559 CHD children and 77,046 non-CHD children (controls). CHD children had a higher need for special education compared to non-CHD children, OR: 2.14 (95% CI: 2.00; 2.28), *p* < 0.001. The odds ratio was also increased when comparing children with a minor CHD to non-CHD children, OR: 1.99 (95% CI: 1.86; 2.14), *p* < 0.001. CHD children exposed to preeclampsia or smoking had a higher risk of receiving special education compared to unexposed CHD children.

**Conclusion:**

We find that school performance is impaired in children born with CHD. This applies to both simple and complex CHD. If a child with CHD was exposed to preeclampsia or maternal smoking this further increased the need for special education.

## 1. Introduction

Children born with congenital heart disease (CHD) have an increased risk of neurodevelopmental difficulties later in life (1–5). The majority of information stems from children born with a complex CHD such as transposition of the great arteries and single ventricle CHD. Executive function and metacognition are often impaired, affecting their school performance (6).

Emerging data suggest that neuropsychological sequelae also occur in children born with simple CHD, although this large group of children is less well studied (1, 5, 7). Since children with simple and uncorrected CHD comprise the vast majority of the overall CHD population, this is an important area to investigate with a potential significant impact for the children, their families and the schools.

There is increasing evidence that multiple factors contribute to the risk of neurobehavioral disability in the CHD population (3). Because operative management strategies can be modified, many early studies and clinical trials of neuroprotective therapies were focused on the perioperative period. However, there is increasing evidence that operative management techniques have a minimal impact on neurodevelopmental outcomes (8). Recent studies have focused on the prenatal conditions such as fetal hypoxia due to an altered circulation (3, 9, 10) and the maternal-fetal environment as potential risk factors for abnormal brain development and adverse neurodevelopmental outcomes. The Barker hypothesis describes how an impaired maternal-fetal environment, like preeclampsia or maternal smoking, has a great effect on postnatal life (11).

Preeclampsia is a pregnancy-related syndrome with origin in abnormal trophoblast function and maternal endothelial dysfunction and it is thought to be a consequence of placental dysfunction and an impaired maternal-fetal environment (12). We know that exposure to preeclampsia increases the risk of neurodevelopmental disorders, and that preeclampsia is overrepresented in children born with CHD (13–17). Whether exposure to preeclampsia affects school performance and the need for special education in children with CHD remains to be investigated. Another factor impacting the maternal-fetal environment is maternal smoking. The impact of maternal smoking on the need for special education also remains unknown.

The primary aim for this study was to evaluate the school performance and the need for special education in a population of all Danish children born with both simple and complex CHD between 1994 and 2012. Our secondary aim was to investigate if exposure to preeclampsia or smoking affects this need for special education.

We hypothesize that children born with both simple and complex CHD have school performance issues and a greater need for special education compared to their healthy peers and we further hypothesize that having been exposed to an impaired maternal fetal environment with preeclampsia or smoking will worsen this outcome.

## 2. Material and methods

### 2.1. Data sources

This study was conducted as a nationwide population-based registry study. All Danish citizens can use the Danish health care system free of charge and it is equally accessible to all. Since 1968 each citizen have held a unique personal identification number ensuring access to healthcare but also linkage between the national registries (18). Data included in this study were obtained using national registries, including: The Danish National Patient Registry (DNPR), The Danish Cytogenetic Central Registry, The Danish Medical Birth Registry, The Family Sociogroup Registry and The Special Education Registry.

### 2.2. Study population

**Figure 1:**
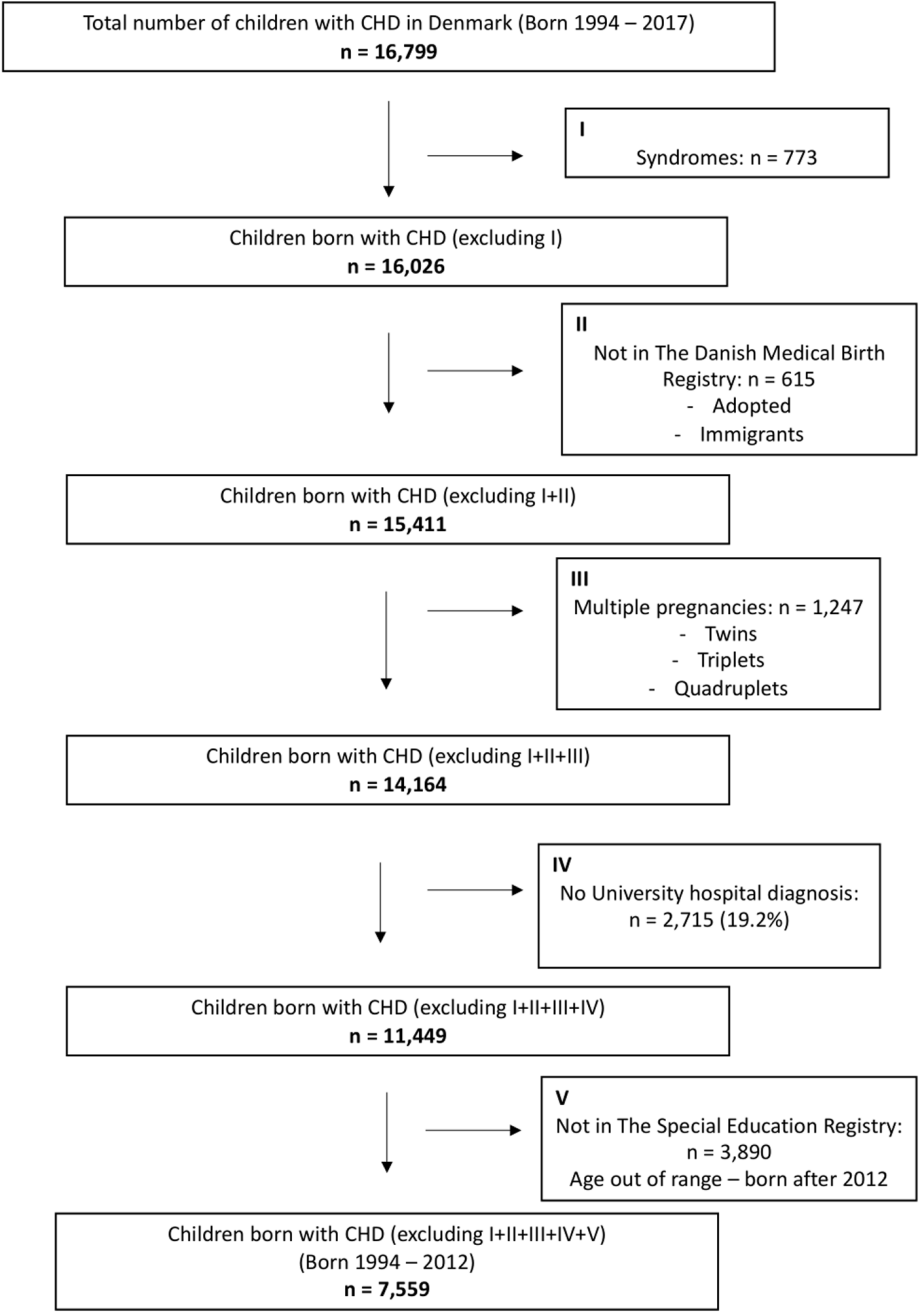
Numbers of children born with CHD included in the study.

The study population has been used in previous studies investigating neurodevelopmental disorders in children with CHD (19). The study population consisted of all children diagnosed with CHD and born between January 1, 1994 to December 31, 2012 in Denmark (Figure 1). A comparison cohort was created of ten randomly drawn age and gender matched individuals without CHD for each CHD patient.

### 2.3. Exclusion criteria: syndromes and non-singletons

#### 2.3.1. Syndromes

Based on data from The Danish Cytogenetic Central Registry, children born with one of the following syndromes were excluded: Trisomy 13, Trisomy 18, Trisomy 21, Turner Syndrome (45,X), Klinefelter Syndrome (47,XXY), DiGeorge Syndrome (22q11 deletion) and Williams-Beuren Syndrome. The Danish Cytogenetic Central Registry is a nationwide register to which all chromosome analyses performed in Denmark since 1960 are reported.

#### 2.3.2. Multiple gestation

Based on data from The Danish Medical Birth Registry non-singletons were excluded. This registry links the mother's information related to her pregnancy and childbirth to the child in question. Individuals not included in The Danish Medical Birth Registry, for example due to adoption from another country or immigration, were likewise excluded. The same exclusion criteria were utilized for the matched control subjects.

#### 2.3.3. Not included in the special education register

The Special Education Register contains information about the need for special education on all children of school age in Denmark from the year 2011 and onwards. Only children listed in this registry were included in the study.

### 2.4. Exposure: CHD diagnosis

Exposure was defined as having a CHD–either simple and complex. Using the DNPR we identified all Danish children diagnosed with CHD between 1994 and 2012. A diagnosis of CHD was defined as ICD-10 codes; DQ20–DQ26. The 10th edition of the ICD has been used since 1994, hence our start of the study period at this point in time. The CHD diagnoses were grouped into either complex CHD or simple CHD in line with previous studies (20). If a child had more than one diagnosis of CHD, we included the most severe diagnoses given at the earliest point in time. To increase the diagnostic validity only children with a CHD diagnosis issued at a University Hospital were included (21).

### 2.5. Outcome: school performance including special education and test results from the Danish national tests

#### 2.5.1. Special education

The outcome was defined as needing special education service in the Danish primary and lower secondary school, which comprises a ten-year period of compulsory basic schooling from 6 years of age until 16 years of age. Schooling from compulsory basic schooling to university level is free of charge in Denmark. Data on the need for special education service was obtained from The Special Education Register. In this register a child needs to have at least 9 h of special education per week in order to be categorised as having the need for special education. We evaluated the need for special education as a dichotomous variable (special education: yes/no). Further, for the children in need of special education we evaluated this need as a continuous variable illustrated as the proportion of time in school spent with special education. This was calculated as: years with special education/total years in school * 100%.

#### 2.5.2. The Danish national tests

The Danish national tests are a nationwide online test system introduced into all Danish primary and lower secondary schools in 2010 (22). The tests are mandatory. Exemption from testing is only possible if school representatives and parents in agreement believe that the student is unable to obtain a useful test result. The tests are designed as adaptive tests and performed in an online self-scoring programme. This means that no teachers are involved in the assessment, ensuring that all students are evaluated by the same standards throughout the country. In this study we included data from the following two subjects: Danish (reading) and mathematics. Danish (reading) was tested in grades 2, 4, 6 and 8, whereas mathematics was tested in grades 3 and 6. We included results based on the students' individual level in the specific subject. Results were reported on a scale from 1 to 6, with 1 being “insufficient performance” and 6 being “excellent performance”.

### 2.6. Additional variables: preeclampsia, smoking, preterm birth and socioeconomic status

#### 2.6.1. Preeclampsia

In the Danish Medical Birth Registry we identified all mothers of the children included in the study. Preeclampsia, eclampsia and HELLP-syndrome in the mother was identified based on DNPR and the following ICD-10 codes: DO11, DO14, DO140, DO141, DO142, DO149, DO15, DO151, DO152, DO159. To ensure correct linkage of mother and the child included in the study the diagnoses of preeclampsia, eclampsia or HELLP-syndrome had to be given maximum 200 days before or 60 days after birth of the child in question. Based on the diagnostic criteria of preeclampsia, eclampsia, and HELLP-syndrome the fetus had to be 20 weeks of gestation or more at the time of diagnosis (23).

#### 2.6.2. Maternal smoking

Information on smoking during pregnancy was obtained with the use of The Danish Medical Birth Registry. Smoking was included as a dichotomous variable (smoking during pregnancy: yes/no).

#### 2.6.3. Preterm birth

Preterm birth (PTB) was considered a potential intermediate factor between CHD and school performance. Preterm birth was defined as being born with a gestational age at delivery of less than 37 weeks. Gestational age (GA) at birth was identified using The Danish Medical Birth Registry.

#### 2.6.4. Socioeconomic status (SES)

SES was considered a potential confounder. SES was obtained based on data from the Family Sociogroup Registry with data on the SES of the family. The parent with the highest income in the family determines the total family SES. The total family SES was used as most children in the study did not yet have individual SES. The family SES was grouped into four groups: Group (1) Business owners and employees with a high income, Group (2) Employees with middle or low income, Group (3) Students and unemployed, Group (4) Others.

### 2.7. Data analysis

Comparison between groups was done using *t*-test for continuous data, and the *χ*^2^ test for binomial data. The difference in need for special education between groups was assessed using logistic regression, allowing adjustment for potential confounders. All statistical tests presumed a significance level of 5%. Data was analyzed using Statistics Denmark's encrypted online data service (Forskerservice), and Stata Statistical Software, release 15 (StataCorp LP, TX).

## 3. Results

The study cohort comprised a total of 7,559 children with CHD (Figure 1). In addition, a total of 77,046 age and gender matched children born without CHD were included.

The distribution of the individual subtypes of CHD can be found in the Supplementary Material S1.

Baseline characteristics of children included in the study are shown in Table 1. No overall differences were observed between children with CHD and no CHD regarding distribution of sex, year of birth nor maternal age. More children with CHD were born preterm and were more often exposed to preeclampsia.

**Table 1:** Baseline characteristics of children included in the study.

	CHD *n* = 7,559 (8.9%)	No CHD *n* = 77,046 (91,1%)	*p*-value
Male sex, *n* (%)	3,848 (50.9)	39,299 (51.0)	0.92
Preeclampsia, *n* (%)	315 (4.2)	1,897 (2.5)	<0.001
Preterm birth: GA < 37 weeks, *n* (%)	1,142 (15.1)	3,485 (4.5)	<0.001
Small for gestational age, *n* (%)	861 (11.4)	9,000 (11.7)	0.45
Maternal age, mean (95% CI)	29.8 (29.6; 29.9)	29.8 (29.7;29.8)	0.65
Smoking during pregnancy, *n* (%)			<0.001
Yes	1,369 (18.1)	12,755 (16.6)	
Missing	830 (11.0)	8,297 (10.8)	
Year of birth, *n* (%)			0.12
1994–1999	1,966 (26.0)	20,294 (26.3)	
2000–2006	3,170 (41.9)	32,933 (42.7)	
2007–2012	2,423 (32.1)	23,819 (30.9)	

Special education was received at some time during their school education by 18.6% of the children born with CHD compared to 9.5% if born with no CHD (Table 2). The numbers and frequencies of children receiving special education stratified on type of CHD, procedure for CHD and exposure to preeclampsia or smoking during pregnancy can be found in Table 2.

**Table 2:** Frequency table of children receiving special education stratified on subgroups.

Special education	Yes *n* = 8,698 (10.3%)	No *n* = 75,907 (89.7%)	*p*-value
CHD, *n* (%)			<0.001
Yes	1,408 (18.6)	6,151 (81.4)	
No	7,290 (9.5)	69,756 (90.5)	
Type of CHD, *n* (%)			<0.001
Simple CHD	1,107 (17.6)	5,183 (82.4)	
Complex CHD	301 (23.7)	968 (76.3)	
No CHD	7,290 (9.5)	69,756 (90.5)	
Operation/procedure for CHD, *n* (%)			<0.001
Yes	586 (23.4)	1,916 (76.6)	
No	822 (16.3)	4,235 (83.7)	
No CHD	7,290 (9.5)	69,756 (90.5)	
Preeclampsia, *n* (%)			<0.001
CHD & PE	76 (24.1)	239 (75.9)	
CHD & no PE	1,332 (18.4)	5,912 (81.6)	
No CHD & PE	224 (11.8)	1,673 (88.2)	
No CHD & no PE	7,066 (9.4)	68,083 (90.6)	
Smoking, *n* (%)			<0.001
CHD & smoking	372 (27.2)	997 (72.8)	
CHD & no smoking	824 (15.4)	4,536 (84.6)	
No CHD & smoking	2,092 (16.4)	10,663 (83.6)	
No CHD & no Smoking	4,178 (7.5)	51,816 (92.5)	

Children with CHD were in significantly greater need of special education in both the primary school (grade 0–5) and in the lower secondary school (grade 6–10). In the primary school 10.8% of children with a CHD received special education compared to 3.4% children with no CHD (*p* < 0.001). In the lower secondary school 24.0% with a CHD received special education compared to 13.6% with no CHD (*p* < 0.001).

Even after adjusting for SES, children born with a CHD had a higher risk of receiving special education compared to children born with no CHD: OR: 2.14 (2.00–2.28), *p* < 0.001 (Table 3). When adjusting for SES and preterm birth, a potential intermediate factor, the odds ratio was found to be OR: 2.04 (1.91–2.18), *p* < 0.001. Further, the odds ratio of receiving special education was also found to be increased when comparing children born with a minor CHD to children born with no CHD. The odds ratio was likewise increased when comparing CHD children who had not undergone surgery to children born with no CHD and likewise when comparing children with a minor CHD who underwent procedure to those with a minor CHO with no procedure (Table 3).

**Table 3:** Odds ratio (OR) of receiving special education.

	OR (95% CI)	*p*
CHD vs. No CHD^a^	2.14 (2.00–2.28)	<0.001
CHD vs. No CHD^b^	2.04 (1.91–2.18)	<0.001
Minor CHD vs. No CHD^a^	1.99 (1.86–2.14)	<0.001
CHD without procedure vs. No CHD^a^	1.83 (1.69–1.98)	<0.001
Major CHD vs. Minor CHD^a^	1.46 (1.26–1.70)	<0.001
Minor CHD with procedure vs. minor CHD with no procedure	1.45 (1.25–1.68)	<0.001

^a^ Adjusted for Socioeconomic Status.

^b^ Adjusted for Socioeconomic Status and Preterm Birth.

When considering only the group of children receiving special education, we found a difference in the amount of special education needed between CHD children and non-CHD. Of the total years spent in school, a minimum of 9 h special education weekly was needed in 53.2% of the years (95% CI: 51.5–54.9) if born with a CHD compared to 45.1% of the years (95% CI: 44.4–45.8) if born with no CHD, *p* < 0.001.

From Table 4 we see that no great difference appears in test results from the Danish national tests between CHD children and non-CHD children of those on need of special education. However, in this group of children receiving special education a large group of children born with CHD are exempt from testing compared to those born with no CHD. This applies both to Danish and Mathematics. The same pattern is not as evident in the group of children not receiving any special education (Table 4).

**Table 4:** Performance in the Danish national tests stratified on the receival of special education.

	Children receiving special education		*p*-value	Children with no special education		*p*-value
	CHD	No CHD		CHD	No CHD	
**Danish**						
Grade (mean, 95% CI)						
2. School year	3.2 (3.0–3.4)	3.3 (3.2–3.3)	0.27	4.1 (4.0–4.1)	4.2 (4.1–4.2)	<0.001
4. School year	2.9 (2.7–3.0)	3.0 (3.0–3.1)	0.04	3.9 (3.9–4.0)	4.0 (4.0–4.1)	<0.001
6. School year	2.9 (2.8–3.0)	3.1 (3.1–3.2)	0.01	4.0 (4.0–4.1)	4.1 (4.1–4.2)	<0.001
8. School year	3.2 (3.0–3.3)	3.3 (3.2–3.3)	0.11	4.2 (4.2–4.3)	4.3 (4.3–4.4)	<0.001
Exempt from testing (*n*, %)						
2. School year	175 (30.3)	416 (13.5)	<0.001	13 (0.5)	78 (0.2)	0.03
4. School year	179 (27.9)	452 (12.0)	<0.001	7 (0.3)	72 (0.2)	0.74
6. School year	190 (27.2)	503 (11.8)	<0.001	10 (0.4)	50 (0.2)	0.01
8. School year	192 (31.1)	502 (13.6)	<0.001	11 (0.5)	101 (0.4)	0.41
Mathematics						
Grade (mean, 95% CI)						
3. School year	3.0 (2.9–3.2)	3.1 (3.1–3.2)	0.09	3.9 (3.8–3.9)	4.0 (4.0–4.0)	<0.001
6. School year	2.9 (2.8–3.1)	3.1 (3.0–3.1)	0.02	3.9 (3.9–3.9)	4.0 (4.0–4.0)	<0.001
Exempt from testing (*n*, %)						
3. School year	166 (27.8)	406 (11.8)	<0.001	10 (0.4)	58 (0.2)	0.04
6. School year	193 (27.5)	534 (12.6)	<0.001	10 (0.4)	54 (0.2)	0.02

Regarding preeclampsia and school performance, we found that CHD children exposed to preeclampsia had a higher risk of receiving special education than CHD children unexposed to preeclampsia: OR: 1.40 (1.07–1.84), *p* = 0.014 (Table 5). The same was found in children born with no CHD (Table 5).

**Table 5:** Odds ratio (OR) of receiving special education if exposed to PE vs. unexposed to PE.

Exposed to PE vs. unexposed to PE	OR (95% CI)	*p*-value
Children born with CHD	1.40 (1.07–1.84)	0.014
Children born with no CHD	1.29 (1.11–1.49)	0.001

Adjusted for Socioeconomic Status. PE, preeclampsia.

## 4. Discussion

In this nationwide population-based cohort study we found that twice as many children with CHD needed special education compared to their healthy peers. This was also true for children with a simple CHD*.* Of those with the need of special education, two-three times as many children with CHD were exempted from school testing. If the mother suffered from preeclampsia or had been smoking during pregnancy, the CHD children were in even higher need for special education. Adjusting for preterm birth modified the risk of needing special education–yet this risk decreased only minimally, suggesting that the total effect is not primarily mediated through preterm birth.

From previous studies, we know that children born with a CHD have a reduced educational achievement (24), and that children born with a complex CHD have a greater need for special education (25, 26). This study points at a substantial school performance issue in a previously neglected group of children born with a simple or uncorrected CHD. We thus found that children born with a simple CHD and children who had not undergone any surgical procedure for their CHD also had a greater need of special education compared to children born with no heart disease.

We found no great difference in the Danish national school test results between children with CHD and non-CHD children–this applied to both the group of children receiving special education and the group of children not receiving special education. However, a large group of children born with CHD was exempt from testing if they received special education. As previously mentioned, exemption from testing was only possible if school representatives and parents in agreement believed that the student was unable to obtain a useful result. Approximately thirty percent of children with CHD receiving special education were considered unable to obtain a useful result. This number was about three times higher in children with CHD compared to children with no CHD, indicating that children born with a CHD and receiving special education might be more challenged than children born with no CHD and receiving special education.

For many years it has been the belief, that the impaired neurodevelopment seen in children with CHD was related to their surgical procedures and medical treatment after birth. However, in this study, we find that even children with CHD who have not undergone surgery have a greater need for special education compared to healthy peers. This indicates that it is not only the treatment of the cardiac defect that results in neurodevelopmental problems. Likewise we found that children born with a simple CHD had a greater need for special education. This is in line with findings in recent studies from our group showing an impaired neurodevelopment in children born with a simple CHD (5, 7) and an increased risk of lifetime psychiatric morbidity and a decreased work participation in young adults with an atrial septal defect (1, 27).

The association between CHD and an impaired neurodevelopment is complex, and the underlying mechanisms are most likely multifactorial. However, the intrauterine environment seems to be an important factor, with intrauterine hypoxia proposed as a leading cause for the impaired cerebral development seen in children with CHD (3, 9, 10). Children born with CHD are more often exposed to preeclampsia *in utero* than those with no CHD (13). Data from our study suggest that preeclampsia further affects the outcome in this already challenged intrauterine environment. We thus found that children with CHD and exposed to preeclampsia have a higher risk of needing special education compared to children with CHD not exposed to preeclampsia, demonstrating that exposure to preeclampsia does affect school performance in children with CHD. Further, we found that nearly twice as many CHD children exposed to maternal smoking received special education compared to those unexposed to smoking.

### 4.1. Limitations

A limitation to this study is that the accuracy of the Danish registries depends on correct diagnostic coding. However, these nationwide registries provide us a unique opportunity to include not only a selection of the population of interest but in fact the entire population (18), in this case all Danish children born with a CHD between 1994 and 2012. To account for inaccurate diagnoses, we only included CHD diagnoses issued at a University Hospital.

Another limitation is that the Special Education Register providing data on special education was established in year 2011. Potentially some children could have received special education prior to this. The lack of knowledge prior to 2011 is the same for children with CHD and non-CHD children, and will most likely underestimate our findings. Further, children born outside Denmark was excluded from this study, which potentially could underestimate our findings.

### 4.2. Conclusion

In conclusion, we find that school performance is impaired in children born with CHD. This applies to both simple and complex CHD. More children with CHD need special education and of these a large group is considered unable to complete the Danish national tests. If a child with CHD is exposed to preeclampsia this increases the risk of needing special education later in life.

In this study we demonstrate that children with CHD, and in particular those exposed to preeclampsia or maternal smoking, are a vulnerable group of children when entering the school system. In order to facilitate them the best possible schooling, we urge clinicians and teachers to be especially attentive to these children.

## Data Availability

The datasets presented in this article are not readily available for legal reasons, this dataset is stored on an encrypted online portal at “Statistics Denmark”. Requests to access the datasets should be directed to Marianne Andresen from Statistics Denmark, MIA@dst.dk.
