# Supplementary material for: School performance is impaired in children with both simple and complex congenital heart disease
Source: Front Pediatr. 2023 Feb 23;11:1073046. doi: 10.3389/fped.2023.1073046 (PMC9995927; doi:10.3389/fped.2023.1073046)
Supplement: Supplementary file 1 [file Table1.docx]

Supplemental material 1: Distribution of the individual types of CHD

|  | CHD children  n = 7,559 |
| --- | --- |
| Minor CHD   - VSD - CoA - Aortic valve disease - Pulmonary valve disease - MV disease - PAPVD - ASD - PDA - Simple Miscellaneous | 6,290 (83.2)  2,108 (27.9)  260 (3.4)  328 (5.1)  661 (8.7)  329 (4.4)  19 (0.3)  1,592 (21.1)  780 (10.3)  30 (0.4) |
| Major CHD   - UVH - TAC - I/HAA - TGA - AVSD - TAPVD - PA - Ebsteins anomaly - TOF - Tricuspid valve disease - Eisenmeger syndrome - Complex Miscellaneous | 1,269 (16.8)  57 (0.8)  38 (0.5)  72 (1.0)  306 (4.0)  253 (3.3)  36 (0.5)  31 (0.4)  48 (0.6)  239 (3.2)  33 (0.4)  39 (0.5)  143 (1.9) |

VSD: Ventricular septal defect, CoA: Coarctation aortae, MV disease: Mitral valve disease, PAPVD: Partial anomalous pulmonary venous drainage, ASD: Arterial septal defect, PDA: Persistent ductus arteriosus, UVH: Univentricular heart, TAC: Truncus arteriosus communis, I/HAA: Interrupted/Hypoplastic aortic arch, TGA: Transposition of the great arteries, AVSD: Artrioventricular septal defect, TAPVD: Total anomalous pulmonary venous drainage, PA: Pulmonary Atresia, TOF: Tetralogy of Fallot
